# Endogenous NHR ligands: metabolomics to the rescue

**DOI:** 10.18632/aging.100676

**Published:** 2014-06-27

**Authors:** Axel Bethke, Frank C. Schroeder

**Affiliations:** Boyce Thompson Institute and Cornell University, Ithaca, NY 14853 USA

Whereas storage of information in biological systems generally relies on large macromolecules, e.g. DNA and RNA, signaling, that is transduction of information, proceeds to a large extent via biogenic small molecules, metabolites of diverse chemical make-up and biosynthetic history. Biogenic small molecules (BSMs) serve as signals within a single cell, as hormones or second messengers between different cells or tissues of one organisms, or as pheromones and quorum sensing signals between individuals of the same or several different species. BSM structures are not only extremely diverse (they may include virtually any structural feature an organic chemist could think of), but their associations with specific signaling roles are often impossible to predict. As a result, the structural and functional annotation of metabolomes has lagged greatly behind advances in genome sequencing and proteomics (Forseth and Schroeder, Curr Opin Chem Biol; 2011).

Nuclear hormone receptors (NHRs) are small molecule ligand-regulated transcription factors that play a central role in metazoan biology and integrate information from many different upstream signaling cascades. NHRs provide one particularly intriguing example for small-molecule-based signal transduction: upstream pathways regulate the expression of a series of biosynthetic enzymes that each impart specific chemical features onto the nascent small-molecule ligand. Therefore, knowledge of the exact structures of NHR ligands provides insight in how upstream signaling controls NHR function. Moreover, the identification of several different ligand structures may reveal different modes of NHR function. The endogenously produced small-molecule ligands of metazoan NHRs are usually of great potency, and their abundance among other metabolites is generally low and tightly regulated. Their low abundance adds to the challenges of characterizing this particular group of BSMs, and as a result the exact structures of the ligands of many NHRs in mammals and the model organisms *C. elegans* (Taubert et al., Mol Cell Endocrinol; 2010) and Drosophila (King-Jones and Thummel, Nat Rev Genet; 2005) have remained unknown or incompletely characterized, presenting a major impediment for the study of NHR function.

The *C. elegans* NHR DAF-12, a homolog to mammalian vitamin D receptor and FXR, plays a central role in lifespan regulation and developmental timing. In the absence of its endogenous steroidal ligands DAF-12 promotes arrest of larval development and entry into a long-lived, highly stress-resistant alternate larval stage called dauer, whereas liganded DAF-12 allows rapid maturation of larvae to reproductive adults (Antebi et al., Genes Dev; 2000, Lee and Schroeder, PLoS Biol; 2012).

The decision between maturation and arrest occurs at the level of transcriptional regulation of several ligand biosynthetic genes downstream of conserved insulin and TGF-β signaling. The last step in DAF-12 ligand biosynthesis is catalyzed by a cytochrome p450 called DAF-9, which converts inactive precursor steroids into the active ligands that bind to DAF-12, thereby promoting reproductive development (Gerisch et al., Dev Cell; 2001). Correspondingly, loss of DAF-9 expression leads to constitutive developmental arrest due to complete abolishment of ligand biosynthesis. Following pioneering work by Antebi and others, Motola et al. used a bold candidate-based approach combined with an array of in-vivo and in-vitro assays to show that two cholestenoic acid derivatives, named Δ^4^- and Δ^7^-dafachronic acid (DA), activate DAF-12 and promote reproductive development in *C. elegans* (Motola et al., Cell; 2006). However, the proposed ligands Δ^4^- and Δ^7^-DA could not be unambiguously detected in *C. elegans*, chiefly because of their low abundance and the vast complexity of the *C. elegans* metabolome, which remains still largely unexplored.

Our recent study aimed to address the question whether Δ^4^- and Δ^7^-DA in fact represent the endogenous DAF-12 ligands and whether perhaps other ligands with unanticipated structural features exist (Mahanti, Bose et al., Cell Metabol; 2014). To overcome the difficulties presented by the ligand's low abundance and the complexity of the metabolome, we employed a recently developed strategy that promises to revolutionize how bioactive small molecules are identified from complex biological systems: comparative metabolomics. This strategy circumvents the need for the isolation of pure compounds via extensive activity-guided fractionation, usually the most arduous part of identifying a new bioactive metabolite. Instead, compound identification relies on comparing high-resolution spectroscopic data sets, for example two-dimensional NMR spectra or HPLC-MS data, from one set of metabolome samples that contain the molecule of interest with a second set of samples that do not contain the molecule of interest, but are otherwise as similar as possible. For identification of the DAF-12 ligands, we applied this concept by comparing the metabolome of wild-type worms with that of *daf-9; daf-12* double mutant worms, which do not produce any ligand due to lack of the biosynthetic enzyme DAF-9. These comparative metabolomic analysis produced several unexpected results: (i) the most abundant DAF-12 ligand includes an unexpected structural element previously known almost exclusively from non-natural, synthetic steroids, (ii) one previously proposed ligand, Δ^4^-DA, was not found in any of the analyzed metabolomes, and (iii) previous hypotheses about the biosynthetic pathways of DAF-12 ligands must be revised. Ultimately, the results of our comparative metabolomic analysis suggest that ligand synthesis is separated into multiple pathways in tissues as distinct as neurons, intestinal cells, hypodermis and spermatheca, producing several chemically distinct ligands. We hypothesize that structural differences between the detected ligands incorporate information beyond the simple presence/absence of a signal, by activating partially distinct downstream cascades. However, available in vivo and in vitro assays have so far not allowed to attribute different functions to the different identified ligand structures.

The chemical space of possible small molecule structures is so vast and diverse that testing synthetic candidate structures, despite many demonstrated successes, may not lead to identification of the actual endogenous signaling molecules, and our work shows that the elucidation of the precise ligand structures is necessary to understand input from upstream signaling pathways. Comparative metabolomics represents a new paradigm that we feel will greatly accelerate the pace of discovery in the area of small-molecule signaling, especially with regard to the functional annotation of the many 1,000, perhaps 10,000 yet uncharacterized metabolites in higher animals, some of which may represent potent and specific nuclear receptor ligands.
